# Redefining the Scholar-Athlete

**DOI:** 10.3389/fspor.2019.00010

**Published:** 2019-08-16

**Authors:** Joseph L. Cross, Bruce W. Fouke

**Affiliations:** ^1^Carl R. Woese Institute for Genomic Biology, University of Illinois at Urbana-Champaign, Champaign, IL, United States; ^2^Department of Geology, University of Illinois at Urbana-Champaign, Champaign, IL, United States; ^3^Department of Microbiology, University of Illinois at Urbana-Champaign, Champaign, IL, United States; ^4^Roy J. Carver Biotechnology Center, University of Illinois at Urbana-Champaign, Champaign, IL, United States

**Keywords:** scholar, athlete, student, athletics, professional skills, academics, identity

## Abstract

Successful Scholar-Athletes are physically, intellectually, and emotionally committed to high-level achievement in both their academic and sport endeavors. This requires development of an integrated skill-set that includes teamwork, a strong work ethic, commitment, leadership, time management, and physical and emotional health. The identity crosses all perceived boundaries of race, gender, ethnicity, sexual orientation, religion, disability, social, and economic status. A nationwide paradigm shift is urgently needed to recognize and tap into these skills for all scholar-athletes, which are the same tools required to succeed in all professions from science and technology to law, medicine, business and the arts. This article addresses the misperceptions and low expectations that much of our society has for the high school and collegiate Scholar-Athlete. While recognizing the efforts of programs that are working to recalibrate the high school athlete's self perceptions, awaken recognition of their own academic potential, and permit them to achieve successful careers and make invaluable professional contributions to society.

## Introduction

According to the National Center for Education and Statistics, ~20.5 million students are projected to enroll in United States (U.S.) high schools in Fall 2017. Nearly eight million of these young people are formally involved in team sports (National Collegiate Athletic Association, 2018). These high school students will herein be referred to as ***Scholar-Athletes***. In this context, the word “Scholar” refers to a student who develops a broad holistic approach to education that includes learning about everything from science and math to art and language. “Athlete” refers to students committed to achieving high performance and success in a wide variety of sports from basketball and soccer to fencing and archery. According to the document “NCAA Recruiting Facts,” ~5–6 percent of the Scholar-Athletes who graduated from high school in 2017 will play NCAA collegiate sports (National Collegiate Athletic Association, 2018).

The purpose of this study is to recognize and awaken academic potential, as well as physical and intellectual perseverance, in today's Scholar-Athletes who seek to achieve successful careers outside of sport and thus make lifelong contributions to society. This review recognizes the need for Scholar-Athletes to tap into skill-sets they already possess, but need to develop, in order to successfully complete their education and attain meaningful and fulfilling careers. NCAA-regulated team sports in the U.S. bring young athletes together on campuses, requiring a basic level of academic achievement while teaching them to socialize, solve problems, resolve disputes, work-hard, gain self-confidence, and understand differing personalities from various cultural perspectives. One might assume that these invaluable traits are fully appreciated, understood and integrated in academic and athletic programs. Yet, in stark contrast, the current higher education system still largely perceives academics and athletics as polarized, unequal, and separate entities (LeWinter et al., 2013). This societal attitude is driving a spirit of virtual animosity between academics and athletics on many U.S. campuses, which is creating a rift that is especially acute and growing in higher education across the U.S. (Beyer and Hannah, 2000). Commonly referred to as “academics vs. athletics,” it is not uncommon for many Scholar-Athletes to feel pressure to prioritize sports over their academic work (Shortell, 2013). As a result, the fundamental integrity of higher education continues to be threatened by students whose sole incentive is to become an elite athlete. This topic directly confronts and evaluates this perceived *status quo*, which is creating a damaging rift between academics and athletics. In reality, both academics and athletics are complimentary and mutually supportive endeavors. The Scholar-Athlete is therefore presented in this topic as a student who possesses precisely the professional skill set needed to eventually excel in everything from science to the humanities.

It is not easy for the scholar-athlete to fully commit to the dual role of scholar and athlete. It takes a person's full dedication for each of those roles. Talent is not only found in sports, but also comes in effective scheduling for classes, study time, work-outs, events, and rest (Axtell, 1991). This ability to juggle everything becomes an asset when the scholar-athlete proceeds toward a career after they are finished playing their respective sport.

**Figure 1:**
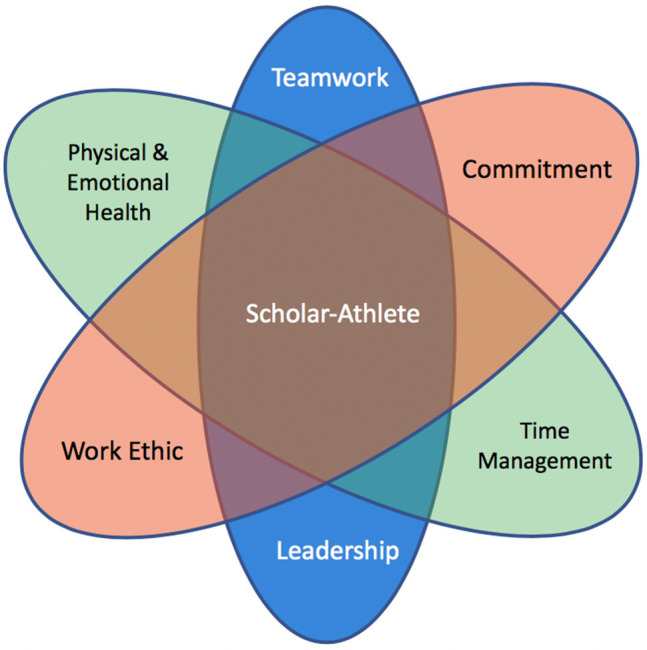
The Scholar-Athlete skill sets that empower young people to achieve successful careers and make meaningful professional contributions to society.

To be successful in career transition, athletes must proactively focus on the importance, awareness, and marketability of transferable skills (McKnight, 2007). The Scholar-Athlete tool kit (Figure 1) includes: (a) *Teamwork*—a person learns to get along with, work with and connect with other people who are different from them in order to achieve a common greater purpose or goal; (b) *Work ethic*—a person develops a strong belief that there is value to working hard to achieve honorable goals, the process of which develops resiliency and strength of character; (c) *Commitment*—a person gains understanding that with every great freedom comes an even greater responsibility, and that commitment is required to bring your endeavors to a successful completion; (d) *Leadership*—a person gains the ability to inspire and constantly teach through their actions and their words; (e) *Time management*—a person must prioritize tasks effectively in order to meet deadlines and achieve worthy goals; and (f) *Physical and emotional health*—a person learns to recognize the importance of a whole and well-balanced lifestyle. The listed skill-set was compiled based on previous research of athletes who identified the most desirable skills and confirmed their existence (Petitpas et al., 1992; Danish et al., 1993; Mayocchi and Hanrahan, 2000; McKnight, 2007; Chalfin et al., 2015). Studies have supported the notion that athletes who capitalize on transferable skills are more likely to experience successful change between athletic and non-athletic settings (Blinde and Greendorfer, 1985). Collegiate coaches and administrators are always searching for an athletic edge, while faculty and admission offices are actively searching for the same type of academic excellence—prowess in the field is not only for sports, it is also for academics which takes many hours of mental agility, study, and hard work, skills not thought of in reference to Scholar-Athletes. The current academic-athletic rift is now in the process of widening on U.S. college campuses and in society as a whole. This contentious mindset starts with a limited and distorted view regarding what young people see as the pathway to success.

## What is a Scholar-Athlete

Advancing the knowledge of what a Scholar-Athlete is, goes beyond sport-minded individuals who want to pursue careers in the sciences, the humanities and all other fields. The Scholar-Athlete's scholastic work, agility of mind, and many hours of studying lends itself toward achievement in any workplace. This professional skill set that the Scholar-Athlete possesses is powerful enough to achieve success in any career or field of study. A Scholar-Athlete is someone who is committed to improving themselves while participating in a sport (Segelken, 2014). This definition crosses all perceived boundaries of race, gender, ethnicity, sexual orientation, religion, and social and economic status.

The Cambrdige Dictionary's (2019) definition of a scholar is a person who has a great knowledge, usually in one subject—the individual studied a great deal in one area. At a fundamental level, all Scholars are in an ongoing quest for knowledge and understanding. Since we are creatures of habit, our daily routines are made up of repetitive practices that automatically produce behaviors for success (Wiest, 2018). If our approaches seem to be working, we keep using them, while if they fail, we stop using them. In science, the process is described as “analogy identifies anomaly” (Winkelmes et al., 2014). Here personal experiences and knowledge provide a comparative standard (“analogy”) against which new unknown experiences and events are evaluated. If new circumstances have never been seen before, then an “anomaly” has been identified that requires adjustment and new rapid learning in order to succeed. As a result, the scholar-athletes themselves define and shape how they are evaluated as different individually in gender, creed, class, and race; but equal as athletes within society (Axtell, 1998).

An Athlete diligently practices the skills needed to obtain the physical and mental “knowledge” required to maximize performance in sport. Much like the traits of a Scholar, the Athlete finds their identity in their daily routine, which includes multiple periods of time set aside to train, practice or compete in their chosen sport. Just as Scholars are careful in how they systematically approach a topic of study, so too does an Athlete carefully consider and study behavioral aspects, such as diet, nutrition, hydration, exercise and performance. The common televised perception of an Athlete is that of a person in an organization including the Olympics and the National Collegiate Athletic Association. Athletes are also people recognized and respected on the streets of our communities and in parks, local schools, recreational clubs, and neighborhood courts. The societal concept of both Scholars and Athletes need to be fundamentally reframed and redefined to be viewed as equivalently respected and understood. These need to become familiar action words used every day to describe an active person who is fully engaged in holistically advancing their personal and professional future.

It is important to note that previous academic research has presented a significantly different definition that is less inclusive and carries socially divisive definitions for Scholars and Athletes, respectively. For instance, Snyder and Spreitzer (1992) define the scholar as a “good student” and one who achieves high scores on tests or even become an honor student in their class. They see Scholars exclusively as those who achieve the highest grades and therefore fall into the socially defined norm of academic prestige and resulting social stratification (Snyder and Spreitzer, 1992). As another example, Axtell (1991) further associates a Scholar as a high achieving student who is older, arguing that true Scholars mature slowly regarding their depth of understanding. Axtell further proports, without evidence, that the social environment of the Scholar is exclusively in the college or university setting, and even here it can only be achieved in graduate school. This redefinition of a Scholar-Athlete links directly to previously established understandings for how individual students establish their own personal identity (Jones and McEwen, 2000). In the present topic, their basic model called Multidimensions of Identity has been applied to Scholar-Athletes with respect to their: (a). Academic Identity; (b). Athlete Identity; (c). Social Identity; and (d). Gender Identity (Jones and McEwen, 2000). This conceptualization draws from understandings of what Athletes have to endure throughout their lives while incorporating the overall complex series of events involved in Scholar identity development. Scholar-Athletes have additional demands imposed by their sports, which create considerable challenges to student life (Jolly, 2008). Research by Schlossberg (1981) identified another theoretical model that can be related to any individual that is going through identity transitioning, such as degree or career shifts, that leads to a modification in one's behavior and relationship. Throughout the complex transitional process, Schlossberg (1981) suggested that the individual's adaptation to the identity transition is dependent on three factors: (a) Biological and social factors specific to the individual, such as gender, age, socio-economic status, culture, and religious beliefs; (b) Situational factors, is the individual in control of the decision to transition or is it forced; and (c) Pre-transition and post-transition environmental factors, does the individual have enough internal and external support from family or outside agencies. Schlossberg's model suggests these three factors influence one's ability to identify and adapt to various life events. Such great awareness of transferable skills by Scholar-Athletes also supports the theoretical research of Danish et al. (1993) Life Development Intervention (LDI) in the process of athletic career transition. The LDI model identifies a similar process specific to athletes and the factors that play into athletic career retirement and the acquirement of transferable skills from sport. Basic principles of the LDI, growth and change, are imperative for human development (Danish et al., 1993). A variety of factors link the Model of Transition (Schlossberg, 1981) and LDI model (Danish et al., 1993) to a process that empowers continual growth while transitioning from sport to degree or career and the implementation of such skills. Awareness of transferable skills leads to an awareness of how such skills were developed.

## Scholar-Athlete: Current *State-of-the-Art*

In U.S. higher education, there is a greater emphasis being placed on the athletic abilities of Student-Athletes and the success of their teams, rather than their success in the classroom (Harrison et al., 2010). College sports have increasingly become a popular form of mass commercial entertainment (Gerdy, 2006). Many elite high school athletes are seen on ESPN signing their national letter of intent, which is an NCAA document committing them to play at a specific institution. The primary purpose of higher education, which is to prepare individuals to make lifelong productive contributions to society, is overlooked far too often. As the popularity and social status of sport continues to rise, middle school and high school institutions are now facing the challenge of addressing an increasing lack of academic productivity among some student groups (Bowen and Levin, 2003). When athletic performance is more important than academic success, cultural perceptions then become more important than educational programs. The high ground needs to be retaken in order to challenge and transform the Student-Athlete's courage and vision for their own future.

For many young people in our neighborhood communities and schools, the path to success is seen solely through athletics (Taylor, 2018). This mindset now shapes how young people view the future. Structural and cultural factors, such as upward economic mobility, gender identity, media and street social interactions have an overwhelming influence on a young person's aspirations and expectations to pursue a professional career in sports (Baker et al., 2006). The present study calls for a basic recasting of these perceptions, including instilling in young people the viewpoints that: (a) all athletes are scholars; (b) most athletes should pursue academics over athletics as their primary future pathway toward achieving professional and economic success; (c) all Athletes should consider their athletic ability as only one component of multiple dynamic dimensions of their identity; and (d) all Athletes must consider themselves active scholarly participants in learning rather than passive uninvolved bystanders in the educational process. Our media and communities are saturated with individuals who have used their trained athletic skill-set to break down barriers of the status quo of race, class, gender as it relates to the Scholar-Athlete to become successful in professions other than sports. A few familiar examples include President Gerald Ford, Earvin “Magic” Johnson, and Shannon Miller. President Ford born July 14, 1913 in Omaha, Nebraska where his mother divorced his father just days after he was born and lived a few years as a single parent. Ford developed a love for football and attended the University of Michigan (1930–1934) where he played center, linebacker, and long snapper for the football team and helped lead the Wolverines to two undefeated seasons and back to back national titles in 1932 and 1933. After college he had an extremely successful career in politics and eventually served as the 38th President of the United States from 1974 to 1977 (Greene, 2019).

Earvin “Magic” Johnson was born August 14th, 1959 in Lansing, Michigan lived in low-income housing with his nine siblings raised by his father Earvin Sr. a General Motors assembly worker and garbage collector, and mother Christine Johnson. Earvin developed a love for basketball at a young age and attended Michigan State University in East Lansing where he led the Spartans to a National Title during the 1978–79 season. Magic was drafted first overall in 1979 and played professional basketball as a point guard for the Los Angeles Lakers from 1979 to 1991 where he won five NBA championships. Since Johnson's (NBA Encyclopedia, 2019) retirement from basketball he has become an advocate for HIV/AIDS prevention, an entrepreneur running a conglomerate called *Magic Johnson Enterprises* with a net worth of $700 million, and is part owner of the Los Angeles Lakers, Los Angeles Dodgers, and Los Angeles Sparks.

Shannon Miller was born March 10th, 1977 in Rolla, Missouri. Even though gymnastics as a sport was not popular amongst youth especially girls, Shannon's parents decided to get her involved at the early age of five and soon had her traveling all over the world. Shannon became a USA Olympic icon in gymnastics. In the 1992 Olympic games in Barcelona, Miller came away with five Olympic medals. Then again in 1996 she was a co-captain of the “Magnificent Seven” for Team USA who won the first Olympic Team Gold for Women's Gymnastics in United States history. After retiring at the age of 19 from Olympic competition, Miller (Wikipedia, 2019a) received her undergraduate degrees in marketing and entrepreneurship from the University of Houston and her law degree from Boston College. In 2010 Miller launched a company called *Shannon Miller Lifestyle: Health and Fitness for Women* that advocates for the health and wellness of women and children. In addition to these types of examples of elite athletes, there are significant stories of innumerable other athletes who used their trained athletic skill-set to break down the barriers of the usual state of opinion in reference to race, class, gender as it relates to the Scholar-Athlete. These individuals have also competed at different intercollegiate levels in which many times is neither seen on television or read about in social media outlets, yet they have effectively applied their athletic skill-set to succeed in professional endeavors outside of their sport.

The Scholar-Athlete is an individual that participates in and fully explores his or her ability to achieve excellence in both academics and a sport. The incredible availability and ever-changing array of technological resources that young people have at their fingertips offer great opportunities for them to do research, explore, and even problem solve. The scholarly interactions experienced by all young athletes prepare them for the entirety of their future lives. Furthermore, scholarly activities motivate and inspire young people (Yazzie-Mintz, 2007). Detachment from scholarly pursuits leads to a lack of motivation, falling behind in work, truancy, teenage pregnancy, caring for a family member and student boredom (Hudson, 2017). This psychological disengagement becomes manifested as resistance to schooling and higher dropout rates (Caldwell et al., 1999). Unfortunately, these feelings of disengagement are commonly passed on from adults to children, resulting in the development of deep-seated resistance to education (Adelman and Taylor, 2011). A further result is the feeling of boredom for all aspects of their personal and professional lives. Misplaced priorities have created barriers to Student-Athlete learning and personal development, and calls for reform (Bedford, 2007). To that end, once a person has become an engaged Scholar, the individual gains the curiosity and motivation required for a fulfilling future life. When instructors adequately engage with students, they begin to recognize the potential benefits of learning through the use of practical issues that relate to the scholar's real experiences and personal spaces of knowledge that have roots in the reality in which they live. Scholars stay interested, learn more, and develop a desire to participate in the subject when techniques are used to involve them in collaborative learning projects (Yazzie-Mintz, 2007). Worldwide there is a growing commitment to best practices that engage students starting in middle school in discussions of scholarly teaching and research (Oseguera, 2010).

## What's Not Working?

Several active education-based intervention programs currently focus on ensuring the academic success of Student-Athletes (i.e., NCAA.org). However, these efforts are being directly diluted and negatively impacted by a multitude of factors in modern society, which include everything from scandals and illegal activities to the legalization of collegiate sports gambling (Maykuth, 2018). For instance, dating from the late 1970s through 1980s the football program at Southern Methodist University (SMU) was under massive scrutiny for repeated violations of NCAA rule (Wikipedia, 2019b). The SMU athletics program was found guilty of operating a private account that lured players and their families to accept scholarship offers to play at the university. As a result of continuous violations over a certain period of time, the NCAA inaugurated a rule change that stiffened repeated violations by athletic departments and referred to it as the “death penalty” (Wikipedia, 2019b). Within the past year there have been similar cases, such as at the University of Louisville, in which FBI reports found that Adidas employees had illegally paid $100,000 to the family of a high-profile basketball recruit (Tracy, 2018). As a result, the University of Louisville's President removed the Athletic Director of 20 years as well as the Hall of Fame head coach of their men's basketball team (Tracy, 2018). There has been a long line of college basketball gambling scandals, a legacy that dates to the early 1950's when seven collegiate institutions (i.e., City College New York, Manhattan College, New York University, Long Island University, University of Kentucky, Bradley University, University of Toledo) and thirty-two players around the country were accused of bribery and conspiracy charges (Figone, 1999; Goldstein, 2003). All were in violation of accepting bribes to insure games were lost by certain point margins. Fast forward 40 years and a similar situation occurred during the 1994–1995 Northwestern University basketball season, in which two Wildcat basketball players were indicted of accepting bribes to fix Big Ten basketball games they played in. The payoff was to insure that the Northwestern basketball team lost by more than the gambling point spread. However, the current state of gambling as it relates to collegiate sports has taking on a new face. According to *USA Today* (December 7, 2017), the Federal Supreme Court has heard oral arguments regarding the nationwide legalization of gambling on collegiate sports. This contradicts the Professional and Amateur Sports Protection Act (PAPSA), which was created 25 years ago to stop the spread of sports gambling in the United States (Legal Informational Institute, 2019).

These societal issues are creating a negative culture that threatens the success of Scholar-Athletes. Young athletes in underserved communities often view their athletic ability as a “ticket out the ghetto,” with sports providing a needed resilience, discipline, financial support and self-esteem for those in poverty situations (Smith, 1988). Furthermore, this destructive mindset permeates into the classroom and all aspects of the educational experience in middle school, high school and college. Furthermore, this perception has a negative influence on how Student-Athletes perceive themselves and how they are represented in the media. What's not working today is the mindset that athletics guarantees financial prosperity through professional sports. The social hyperbole and media coverage of wealth and scandals further conflates and cements the perceived linkage between the aspirations of the young athlete and the popularity of the professional athlete. This is a contributing factor in the degeneration and general disrespect being perpetuated by society on Scholar-Athletes. It is unfair to our Scholar-Athletes to place pressure on them to work hard toward a professional athletic career. To have this type of unrealistic and unfounded goal to play professional sports drains and derails the potential realistic opportunities available from other career options (Lomax, 2000). The fact is that far too many young athletes continue to view professional athletics as an attainable lifestyle for them, and believe that they are “Going Pro,” despite the overwhelming evidence that attaining a career as a professional athlete has a low chance of happening (National Collegiant Athletic Association, 2019).

There are many studies that estimate the probability of high school students competing in professional sports and college students participating in professional sports (National Collegiant Athletic Association, 2019). In both cases, those percentages are very low raging from as high as 9 percent to as low as 0.5 percent, depending on the sport (NCAA.org, 2019). In contrast, the likelihood of an NCAA athlete earning a college degree is significantly greater, with graduation success rates reaching 86 percent (NCAA, 2019a). Yet despite these statistics, young athletes continue to foster the unreasonably high expectation that they can actually become a professional athlete (Harrison et al., 2000). Unbelievably, youth today believe that there is a greater chance of them achieving a professional sports career than obtaining a college degree (NCAA, 2019b). As a result, young athletes fail to devise an alternative plan in the event they do not achieve success in their athletic pursuits. This failure becomes magnified in their perceptions of themselves and their future career potentials and opportunities (Brewer et al., 1993).

The reality is that athletes in high profile sports, such as football and basketball identify more closely with successful athletic achievement rather than occupational career success (USA Today, 2019). A number of prominent scholars warn of the detrimental effect of having the single-minded obsession of sports fame (Harrison, 2002). There are stigmas and labels that athletes attach to these images that are part of their perception of success. These types of deficit perspectives are common amongst Student-Athletes, and these threaten a Student-Athlete's ability to choose a major in college (Oseguera, 2010). Athletes view these deficits as major factors when trying to balance their academic, athletic, and social areas of life. As a result, many Student-Athletes never even consider a degree in science, technology, engineering and mathematics (STEM). One of the most fundamentally important ongoing concerns facing Scholar-Athletes is their physical health and strength, and understanding how their nutritional choices affect the performance of their bodies. As a result, there is a direct intrinsic linkage between Scholar-Athletes and their knowledge of health sciences. Student-Athletes have distinct experiences from non-athlete students at the college level (Comeaux, 2010). Factors that influence their choice of a major include the class workload and frequency of daily homework, team practice each day of the week, study table requirements, mentoring by academic advisors, athletic meetings, weight training, athletic conditioning, treatment of injuries, game-day competition, travel, coaching expectations, media interviews, social life, and family. As noted by Comeaux and Harrison (2011), student-athletes experience an institution's social community through engagement with teammates and peers, coaches, faculty, and other on- and off-campus activities. In addition to this, their academic experiences include tutoring sessions, classroom lectures, and faculty office hours (Comeaux and Harrison, 2011). From the Athlete's viewpoint this begs the question: “Which major offers the path of least resistance but greatest economic success?” Studies have indicated that Athletes believe that economic success through sports is a more realistic possibility than any other avenue to success (USA Today, 2019). In turn, this unfounded faith in sports as the best road to success has a negative impact on the Student-Athlete's academic performance. The Student-Athlete who values sport as the most probable means to future economic success devote all their effort to the sport and neglect opportunities for educational achievement and intellectual development (Coach&AD, 2015; Perry, 2017).

## What is Working?

What has been working are the past and present institutional programs that are investing to create a new mindset among Student-Athletes in terms of their perceptions about education, sport and career ambitions. One such institutional program, called the Bridge and Transition program established in 1986 at the University of Illinois Urbana-Champaign campus, was created to address the low graduation rate amongst football players during that time (Cross, 2017). It was designed to provide assistance to a select group of underrepresented students, in which a designated number of football and basketball players were included. The Bridge component of the program was a 6-weeks summer program that provided 50 students with academic and career counseling, extensive academic support, personal development skill training, enrichment activities, and participation in skill-building and academic orientation curricula specifically designed for each cohort of students (Cross, 2017). The Transition component of the program served those involved in Bridge and additional underrepresented students who were Freshmen and Sophomores who had not declared a major. These students received the needed support, advice, and encouragement to be academically successful at the University of Illinois at Urbana-Champaign. The football graduation rate went from 22% in 1986 to 90% from its beginning in 1986 to its end in 2009 (Cross, 2017). The program was nationally known for its excellence in student academic achievement, retention and graduation rates. Yet despite this success, in the summer of 2009, the Bridge program was discontinued. No matter the value and success of the program, the Bridge program fell amongst several governing decisions the University needed to make as a result of tax cuts and financial priorities. How an institution chooses to share its resources or revenues among departments is reflective of what that institution holds dear in its culture.

CHAMPS/Life Skills Program (Challenging Athletes' Minds for Personal Success) is an ongoing institutional initiative created in 1991 by the NCAA to invest in the mindset of the Student-Athlete. The CHAMPS/Life Skills Program was not created to make sure athletes remained academically eligible or to keep them from violence or promiscuity but is committed to the total development of Student-Athletes, which equips them with valuable life skills that are useful during the college experience and after graduation (www.NCAA.org, 2018). Presentations and workshops are comprised of five commitment areas: academic excellence, athletic excellence, personal development, service to campus and communities, and career development. In collaboration with the NCAA national office the CHAMPS/Life Skills Program is recognized within all 1,200 NCAA institutions (NCAA, 2019c). Big change begins with small commitments and engagements. Programs, such as the Bridge and Transition program, as well as the CHAMPS/Life Skills Program, create a dynamic positive branding for the future new definition of the Scholar-Athlete.

The Scholar-Baller Program (Scholarballer.org, 2011), established in 1995, is a creative approach to recognize and reward academic achievement by student-athletes (Scholarballer.org, 2011). The program directly addresses the challenges of balancing participation in sports with academic progress toward attaining a degree. The aim is to send a message to young athletes that success should be equally strived for in both athletics and academics (Scholarballer.org, 2011). This movement seeks to integrate education, sport and hip-hop as a way to motivate athletes in sport and in the classroom (Scholarballer.org, 2011). College athletic departments can formally engage by incorporating six basic Scholar-Baller Program principles, which include identity, competitive spirit, purpose, vision, mission, and goals. There are many universities across the nation using the Scholar-Baller Program curriculum as a compliment to their CHAMPS/Life Skills Programs (NCAA, 2019c).

## The Future

The vision for the future is to seek out academic intervention programs no later than middle and high school that can directly connect the Scholar-Athletes universal skill-sets of teamwork, work ethic, commitment, leadership, time management, emotional and physical health with solving some of the planets more complex problems. These related initiatives developed by institutions and community outreach efforts can be an excellent marketing platform to reposition the direction of Scholar-Athletes. These types of initiatives and media platforms help to draw attention to the academic performance of Scholar-Athletes while still allowing the players to demonstrate their athletic skills. The concept is to continue having athletes strive to attain high performance in their chosen sport, no matter the age, while integrating and nurturing academic research and knowledge in these same areas. It is critical that teachers, instructors, faculty, and administrators team up with today's Scholar-Athlete with these goals in mind. Then, a young person sees and understands these connections that are committed to the Scholar-Athlete life style. By bringing those two identities together, a student who participates in an impactful manner to both academic research and sport becomes the definition of a Scholar-Athlete. The present study advances this perspective as the pathway for successfully redefining the Scholar-Athlete, which will integrate media with educational institutions and draw meaningful connections between education, sport and popular culture.

Obvious first steps include engaging in athletics with specific interesting academic achievements. Examples include: (a) allowing students access to further education to receive college and even graduate diplomas; (b) guiding students to build character by representing their university through competition; (c) nurturing collaboration between teammates to solve problems; and (d) instilling a sense of drive in students in seeing their goals completed. Comeaux and Harrison (2011) define academic success as matriculation and graduation from a program of study. The goals of matriculation include ensuring that Scholar-Athletes integrate successfully into the academic, social and athletic systems of college, complete their course requirements in a specific degree program, and achieve their educational objectives (Comeaux and Harrison, 2011). Because participation in athletics and academics are important in the reward structure of schools and community, there are indeed social expectations inherent in the two roles. Society expects certain attitudinal and behavioral characteristics to be associated with these roles. Thus, if an individual is both a Scholar and an Athlete, we would anticipate that the attitudinal and behavioral differences would vary depending on the individual's degree of involvement in the two roles. Coleman (1961) argues that scholar and athletic participation are positively correlated with having positive self-esteem. He further suggests that the role of scholar and athlete provide societal payoffs in the social structure and are important for the development of adolescent to adulthood. Conventional societal norms should encourage students at an early age to see the value of being a Scholar-Athlete and development of the integrated skill-sets as the key to success.

## Scholar-Athlete Camps

To dynamically instill this vision of the future, a new project called Scholar-Athlete Camps were introduced summer 2018 in partnership between the Carl R. Woese Institute for Genomic Biology (IGB), Kinesiology & Community Health and the Division of Intercollegiate Athletics (DIA) departments at the University of Illinois at Urbana-Champaign. Each camp is tailored to a specific sport, combines cutting-edge concepts in food, nutrition, hydration, the human microbiome, muscle strength and overall fitness. Importantly, all of these concepts are integrated with ideas at the forefront of research dedicated to the search for life throughout the cosmos by the NASA Astrobiology Institute. Campers range in age from elementary school through high school and give athletes their first glimpse of what life is like as a Scholar-Athlete at the collegiate level (Fighting Illini Sports Camps Clinics, 2018). However, these cutting-edge camps are unique in their integrative reach, as well as the strong cooperation between academic and sports entities at a major land grant research university campus.

Scholar-Athlete Camp sessions focused on a variety of topics, including the following:

- *Nutrition and Hydration*—Optimize eating and water intake habits, nutrition, and learn the importance of hydration;
- *The Science Behind Training*—Measure fitness using heart rate, temperature, vertical jump, and maximum amount of oxygen that a person can utilize during exercise (VO_2_ max) to maximize performance and apply scientific measurements to athletic performance.
- *Exercise and Sport in Space*—Evaluation of human activity, health and performance at low or zero gravity to predict how a sport and training regimen would be modified to be played *en route* to another planet.
- *Tree of Life*—Commemorates Carl R. Woese' discovery of the third domain of life, Archaea, in the 1970s. Understanding where all life forms originate from in the three domains of life Bacteria, Archaea and Eukaryotes. Giving meaning to how scientist show how all living organisms are connected.
- *The College Experience*—Learn about life as a college student with Illinois student-athletes, faculty, students, and staff (Fighting Illini Sports Camps and Clinics, 2015).

The middle and high school female and male students who attend the camps come from across the US and other countries. The Scholar-Athlete Camps initiative directly addresses the nationwide need to engage this unique group in innovative, multi-disciplinary research. In so doing, it allows them to utilize and further develop skills, such as teamwork, work ethic, commitment, leadership, time management, and physical and emotional health. As emphasized previously, this professional skill set that the Scholar-Athlete possesses is powerful and is required to achieve success in any future career or field of study. It represents the first experience for most Scholar-Athletes of seeing these intimate connections between the academic and athletic worlds in which they live. In so doing, these camp sessions serve as a model that other institutions can follow. However, the engaged commitment of key academic and athletic partners on a campus are required to: (a) expose participants to integrative areas in science and thus increase awareness of future career and college possibilities; (b) build teamwork and laboratory inquiry skills in a variety of disciplines; and (c) allow students to directly experience ongoing cutting-edge research that can be brought back to their home schools. The ultimate goal is to capture the imagination and creativity of the campers, open entirely new ways for them to think about sport and science, and fundamentally enhance their perspective regarding what the pursuit of higher education is about. Connecting Scholar-Athletes in liberating activities that will help them develop critical and analytical skills (Morrell, 2002). This approach is unique and somewhat startling compared to how effective outcomes are usually expressed to student athletes. This framework also has the benefit of creating a significantly more in-depth and genuine recruiting environment for interactions between coaches and students during recruitment and eventual success and retention once on campus. Furthermore, this provides an opportunity for enhanced feedback from academics to coaches regarding the attitude, behavior, and leadership-skills of the student athletes when in an academic setting. The Scholar-Athlete Camps offer but one example of many academic support-oriented initiatives that can be established at universities across the nation to assist Scholar-Athletes.

## Summary

Scholar-Athletes have the potential to be physically, intellectually and emotionally committed to high-level achievement in both their academic and sport endeavors. Their success requires development of an integrated skill-set that includes teamwork, a strong work ethic, commitment, leadership, time management, and physical and emotional health. The identity crosses all perceived boundaries of race, gender, ethnicity, sexual orientation, religion, disability, social, and economic status. A nationwide paradigm shift is urgently needed to recognize and tap into these skills, which are the same skills required to succeed in all professions from science to the humanities. Another important result of this Scholar-Athlete grass root initiative will be to revolutionize the content and efficacy of the recruiting process that will validate their personal interests both athletics and academics. A Scholar-Athlete is someone who is committed to improving themselves while participating in sport. Moving forward, the words Scholar and Athlete could much more effectively be viewed as equivalent, respected and well-understood terms to describe an active person who is holistically engaged in advancing their personal and professional future. This innovative yet practical mindset for the Scholar-Athlete, which currently is absent in many sectors of our society, will invoke powerful and effective change now and for future generations.

## Author Contributions

All authors listed have made a substantial, direct and intellectual contribution to the work, and approved it for publication.

### Conflict of Interest Statement

The authors declare that the research was conducted in the absence of any commercial or financial relationships that could be construed as a potential conflict of interest.
